# Endothelial lipase genetic polymorphisms and the lipid-lowering response in patients with coronary artery disease on rosuvastatin

**DOI:** 10.1186/s12944-016-0295-3

**Published:** 2016-09-06

**Authors:** Gaojun Cai, Bifeng Zhang, Ganwei Shi, Weijin Weng, Liping Yang, Sheliang Xue

**Affiliations:** 1 Department of Cardiology, Wujin Hospital Affiliated to Jiangsu University, Changzhou, Jiangsu Province China; 2 Department of Pathology and Molecular Medicine, McMaster University, Ontario, Canada

**Keywords:** Endothelial lipase, Coronary artery disease, Single nucleotide polymorphism, Rosuvastatin

## Abstract

**Background:**

Endothelial lipase (EL) plays an important role in the regulation of lipid metabolism by reducing the high density lipoprotein cholesterol (HDL-C) levels and inducing the macrophages to take up native low density lipoprotein cholesterol (LDL-C). Our purpose was to investigate the impact of *EL* genetic polymorphisms on the lipid-lowering effects of rosuvastatin in Chinese coronary artery disease (CAD) patients.

**Methods:**

One hundred twenty-one unrelated CAD patients, who underwent the treatment with rosuvastatin (10mg/day) for four to eight weeks, were enrolled in this study. Before and after treatment, serum lipids levels were measured. Genotypes of *EL* 2037T/C and 2237 G/A polymorphisms were detected by polymerase chain reaction-restriction fragment length polymorphism (PCR-RFLP) method.

**Results:**

Patients with *EL* 2037C allele (CC + CT) had significantly lower LDL-C levels than those with TT genotype (CC + CT: 2.60 ± 0.74 mmol/l; TT: 2.90 ± 0.87 mmol/l; *P* = 0.047), before rosuvastatin treatment. No significant differences between baseline lipid levels and the *EL* 2237G/A genotypes were observed. After treatment with rosuvastatin, total cholesterol (TC), high triglyceride (TG) and LDL-C levels decreased from baseline, on average, by 23.09 % (4.59 ± 0.96 mmol/l to 3.47 ± 0.83 mmol/l), 6.36 % (2.01 ± 1.18 mmol/l to 1.68 ± 1.16 mmol/l), 32.48 % (2.77 ± 0.83 mmol/l to 1.79 ± 0.62 mmol/l), respectively (all *P* < 0.05) in all patients. While changes in HDL-C levels did not reach statistical significance. No significant effects of *EL* 2037T/C or 2237G/A polymorphism were observed on lipid-lowering effects of rosuvastatin.

**Conclusions:**

*EL* 2037T/C and 2237 G/A polymorphisms might not affect the lipid-owing effects of rosuvastatin in Chinese CAD patients.

**Electronic supplementary material:**

The online version of this article (doi:10.1186/s12944-016-0295-3) contains supplementary material, which is available to authorized users.

## Background

Epidemiological data show that coronary artery disease (CAD) will remain one of the leading causes of morbidity and mortality up to 2030. About 7.3 million people worldwide die from CAD annually, according to the report from World Health Organization (WHO) [1].

Dyslipidemia, including high total cholesterol (TC), high triglyceride (TG), high low density lipoprotein cholesterol (LDL-C), and/or low high density lipoprotein cholesterol (HDL-C), is one of the important risk factors for CAD [2, 3]. So in order to prevent and treat CAD, it is most important to decrease the TC, TG and LDL-C levels. Statin, which can reduce the LDL-C levels and enhance the HDL-C levels, is the most important class of the lipid-lowering drugs to prevent and treat CAD [4, 5].

Pharmacogenetic studies have revealed that single nucleotide polymorphisms (SNPs) might influence the lipid-lowering effects of statins [6]. The genetic difference leads to the different lipid-lowering effects of statins in individual patient [7–9]. The results from a meta-analysis revealed that the *ABCB1* C3435T polymorphism might be associated with the lipid-lowering effects of statins in patients with hypercholesterolemia. The LDL-C lowering effects in patients with TT genotype were more significant than those in patients with CC genotype [7]. Li J et al. [8] concluded that the cholesterol ester transfer protein *Taq*IB polymorphism affected the lipid-lowering effects of atorvastatin in CAD patients. After three months of treatment with atorvastatin, the TG levels in B2B2 patients was lower than that in B1 carriers (*P* = 0.009). However, Fu Q et al. [10] found that the *SLCO1B1* polymorphisms were not associated with lipid-lowering effects of atorvastatin and simvastatin in Chinese patients.

The endothelial lipase (EL), which was discovered by Hirata et al*.* [11] in 1999, belongs to the lipase family. Previous studies have revealed that the over-expression of EL in human Apo-AI transgenic mice led to a large reduction in the HDL-C levels. Compared with wild type mice, the scavenger receptor class BI (SR-BI) and ATP-binding cassette transporter A1 (ABCA1)-mediated cholesterol efflux changed greatly in mice with over-expression of EL [12]. It is well recognized that the serum EL concentration was closely associated with cholesterol levels, especially HDL-C levels, in different populations, and the risk of CAD [13]. In vivo, EL expression could be inhibited by statin via Toll-like receptors-4 [14]. The further studies found *EL* gene polymorphisms might affect the serum cholesterol levels, and was associated with the CAD risk [15–17]. However, it is not known whether *EL* polymorphisms can affect the lipid-lowering effects of statins. Therefore, we performed this study to determine the impact of *EL* 2037 T/C (rs3744843), 2237 G/A (rs3744841) genetic polymorphisms on the lipid-lowering effects of rosuvastatin in Chinese CAD patients.

## Methods

### Subjects

A total of 121 CAD patients (87 males and 34 females) with or without hyperlipideamia, randomly selected from our previous hospital based case-control study, were enrolled in the present study. All subjects were selected from the Department of Cardiology of Wujin Hospital affiliated to Jiangsu University between February 2009 and May 2015. Diagnosis of CAD was confirmed according to the WHO criteria. All of the patients had coronary angiography examination. Patients with thyroid disease (hyperthyroidism or hypothyroidism), malignant disease, renal or hepatic disease were excluded.

All patients entering this study were treated with 10mg rosuvastatin daily for four to eight weeks. None of the patients underwent statin treatment for at least one month before entry into this study or took any other lipid-lowering drugs during this period. Before and after treatment, the lipids levels were measured. When the LDL-C levels decreased below 1.80 mmol/l or more than 50 % compared with the baseline LDL-C levels, we seemed the lipid-lowering treatment attaining the guideline levels. To explore the side effects of rosuvastatin, the alanine aminotransferase (ALT) and creatine kinase (CK) levels were detected after treatment.

### Detection of lipid profiles

After overnight fasting, blood was collected from each patient. Serum TC, TG, HDL-C and LDL-C were measured by methods of oxidase. All of the biochemical indexes were analyzed by automatic biochemical analyzer (Olympus AU5400).

### Genotyping of *EL* polymorphisms

The procedure of genomic DNA extraction was according to our previous method [18]. Briefly, the genomic DNA was extracted from peripheral blood by standard phenol-chloroform method and was stored at -70 °C until use.

Genotyping of the *EL* 2037 T/C and 2237 G/A polymorphisms were performed by polymerase chain reaction (PCR) followed by restriction fragment length polymorphism (RFLP) analysis. Amplification of the fragment was performed by using the sense 5′-GTT ACT GCT GAG GAC CCA C-3′ and the antisense 5′-TAG AAA TCC CAA CTC CAC TG-3′ (which were synthesized by Takara Biotechnology, Dalian, Co., Ltd.) for these two polymorphisms. The PCR reactive conditions were: denaturation for 5 min at 94 °C, followed by 35 cycles at 94 °C for 15 s, 58 °C for 30 s, 72 °C for 30s and 72 °C for 10 min. The PCR product was digested with restriction enzyme *Sma I* for 2037 T/C and *BseD I* for 2237 G/A at 55 °C overnight. The digested PCR product was separated by 1.5 % agarose gel electrophoresis and visualized under ultraviolet light. To confirm the genotyping results, a part of PCR products was sent for sequencing (Genewiz, Suzhou, China).

### Statistical analysis

All statistical evaluations were analyzed by using SPSS software (version 17.0, SPSS Inc., Chicago, Illinois, USA). Comparison of continuous variables, which were represented as means ± standard deviation (SD), was performed by using independent-sample *t*-tests or analysis of variance (ANOVA). Paired Student’s *t* test was used for comparing the lipids differences before and after rosuvastatin treatment. The genotype and allele frequencies were counted directly. Qualitative variables were reported as frequencies and percentages, and evaluated by Chi-square test. The genotype distribution for the Hardy-Weinberg equilibrium (HWE) was also evaluated by Chi-square test. A *P* value less than 0.05 (2-tailed) was considered as statistical significance.

## Results

### Baseline characteristics

The baseline clinical characteristics and lipid levels of the present study are shown in Table 1. One hundred twenty-one patients with CAD were enrolled in this study. Of the 121 patients (age range: 39–82 years; mean age: 62.17 ± 9.71 years), 87 (71.90 %) were males. The percentages of smoking, essential hypertension and diabetes mellitus were 34.71 %, 71.90 % and 24.79 %, respectively.

**Table 1:** Clinical characteristics and lipids levels of patients before treatment

Parameters	*n* = 121
Age (years)	62.17 ± 9.71
Male [*n*(%)]	87 (71.90%)
Smoking [*n*(%)]	42 (34.71%)
EH [*n*(%)]	87 (71.90%)
DM [*n*(%)]	30 (24.79%)
TC^a^ (mmol/l)	4.59 ± 0.96
TG (mmol/l)	2.01 ± 1.18
HDL-C (mmol/l)	1.07 ± 0.30
LDL-C (mmol/l)	2.77 ± 0.83
2037 T/C (rs3744843)	
TT [*n*(%)]	68 (56.20%)
CT [*n*(%)]	47 (38.84%)
CC [*n*(%)]	6 (4.96%)
2237 G/A (rs3744841)	
GG [*n*(%)]	46 (38.02%)
GA [*n*(%)]	60 (49.59%)
AA [*n*(%)]	15 (12.40%)

*N* number, *EH* essential hypertension, *DM* diabetes mellitus, *TC* ^a^ total cholesterol, *TG* triglyceride, *HDL-C* high density lipoprotein cholesterol, *LDL-C* low density lipoprotein cholesterol; Continuous variables were represented as means ± SD

### Comparison of lipids levels before and after treatment

**Fig. 1:**
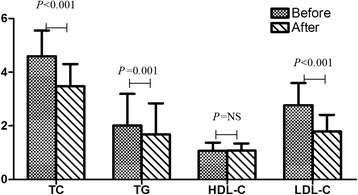
Comparison of lipids levels before and after rosuvastatin treatment in 121 patients with CAD. TC, total cholesterol; TG, triglyceride; HDL-C, high density lipoprotein cholesterol; LDL-C, low density lipoprotein cholesterol; Unit of TC, TG, HDL-C and LDL-C is mmol/l; NS: no statistic; Data are shown as mean ± SD

After treatment with rosuvastatin, TC, TG, and LDL-C levels decreased from baseline, on average, by 23.09 % (from 4.59 ± 0.96 mmol/l to 3.47 ± 0.83 mmol/l), 6.36 % (from 2.01 ± 1.18 mmol/l to 1.68 ± 1.16mmol/l), 32.48 % (from 2.77 ± 0.83 mmol/l to 1.79 ± 0.62 mmol/l), respectively (all *P* < 0.05) in all patients (Fig. 1). However, changes in HDL-C levels did not reach statistical significance (from 1.07 ± 0.30 mmol/l to 1.08 ± 0.26 mmol/l, *P* = 0.815).

### The clinical characteristics of patients stratified by *EL* genotypes

The frequency of the *EL* 2037 C variant allele was 24.38 % in CAD patients, and the genotypic distribution was 4.96 % (*n* = 6) for the CC genotype, 38.84 % (*n* = 47) for the CT genotype, and 56.20 % (*n* = 68) for the TT genotype. For *EL* 2237 G/A, the frequencies of the GG, GA and AA genotypes were 38.02, 49.59, and 12.40 %, respectively. The genotypic distributions of these two polymorphisms were both in accordance with HWE (for 2037 T/C, *P* = 0.35; for 2237 G/A, *P* = 0.50).

The baseline characteristics of patients stratified by *EL* genotypes are shown in Table 2. For *EL* 2037 T/C, patients with C allele (CC + CT) had significantly lower LDL-C levels than those with TT homozygous genotype (CC + CT: 2.60 ± 0.74 mmol/l; TT: 2.90 ± 0.87 mmol/l; *P* = 0.047), before rosuvastatin treatment. For *EL* 2237 G/A, no significant differences in baseline lipid levels between the genotypes were observed.

**Table 2:** The baseline lipid profiles for *EL* 2037 T/C and 2237 G/A genotypes in CAD patients

SNPs	*N*	TC^a^ (mmol/l)	TG (mmol/l)	HDL-C (mmol/l)	LDL-C (mmol/l)
2037 T/C					
TT	68	4.74 ± 1.00	1.98 ± 1.17	1.08 ± 0.25	2.90 ± 0.87
TC	47	4.47 ± 0.88	2.08 ± 1.27	1.09 ± 0.36	2.64 ± 0.74
CC	6	3.92 ± 0.85	1.91 ± 0.57	0.82 ± 0.18	2.32 ± 0.78
TC + CC	53	4.41 ± 0.89	2.06 ± 1.21	1.06 ± 0.36	2.60 ± 0.74^*^
2237 G/A					
GG	46	4.63 ± 1.08	1.81 ± 0.95	1.04 ± 0.28	2.87 ± 0.93
GA	60	4.60 ± 0.85	2.21 ± 1.33	1.11 ± 0.33	2.69 ± 0.75
AA	15	4.45 ± 1.05	1.85 ± 1.16	1.03 ± 0.24	2.81 ± 0.80
GA + AA	75	4.57 ± 0.89	2.14 ± 1.30	1.09 ± 0.32	2.71 ± 0.76

*SNPs* single nucleotide polymorphisms, *N* number, *TC* ^a^ total cholesterol, *TG* triglyceride, *HDL-C* high density lipoprotein cholesterol, *LDL-C* low density lipoprotein cholesterol

^*^ *P* < 0.05 compared with TT group

### Association between *EL* 2037 T/C and 2237 G/A polymorphisms and the lipid-lowering effects of rosuvastatin

For *EL* 2037 T/C polymorphism, TC and LDL-C levels were significantly reduced in all the genotypes compared with before treatment (Additional file 1: Table S1). However, the HDL-C levels did not change in any of the genotypes. TG levels decreased significantly in subjects with TT and TC genotypes.

For *EL* 2237G/A polymorphism, compared with the baseline levels, TC, and LDL-C levels significantly decreased in all of the genotypes. In addition, the TG levels decreased in GA genotype (*P* < 0.001). Conversely, the concentration of HDL-C did not change significantly in any of genotypes.

**Fig. 2:**
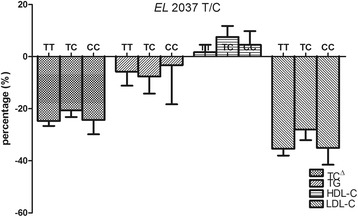
Comparison of percentage changes from baseline in lipid levels among the *EL* 2037 T/C genotypes. TC^Δ^, total cholesterol; TG, triglyceride; HDL-C, high density lipoprotein cholesterol; LDL-C, low density lipoprotein cholesterol; Data are shown as mean ± standard error

**Fig. 3:**
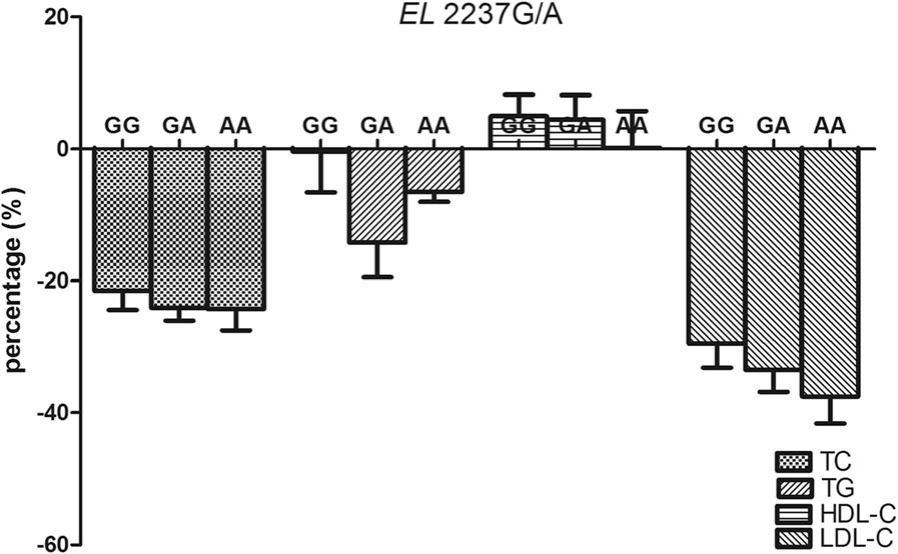
Comparison of percentage changes from baseline in lipid levels among the *EL* 2237 G/A genotypes. TC, total cholesterol; TG, triglyceride; HDL-C, high density lipoprotein cholesterol; LDL-C, low density lipoprotein cholesterol; Data are shown as mean ± standard error

The effects of *EL* 2037 T/C and 2237 G/A polymorphism on the lipid-lowering response to rosuvastatin are shown in Figs. 2 and 3. The changes of lipids were presented as percentage. No significant effects of *EL* 2037T/C or 2237G/A polymorphism were observed in lipid-lowering effects of rosuvastatin.

### *EL* genetic polymorphisms and the percentage of patients attaining the guideline LDL-C levels

In the present study, the percentage of patients attaining the guideline LDL-C levels was 61.98 % (75/121). The percentage in *EL* 2037 CC, CT, TT genotypes was 58.82 % (40/68), 63.83 % (30/47) and 83.33 % (5/6), respectively. In addition, the percentage in *EL* 2237 GG, GA, AA genotypes was 52.17 % (24/46), 66.67 % (40/60) and 73.33 % (11/15), respectively. No significant difference was found between *EL* genetic polymorphisms and the percentage of patients attaining the guideline LDL-C levels (for 2037 T/C: χ^2^ = 1.517, *P* = 0.468; for 2237 G/A: χ^2^ = 3.257, *P* = 0.196).

### *EL* genetic polymorphisms and side effects of rosuvastatin

After treatment with rosuvastatin, the alanine transaminase (ALT) in three cases increased more than three times of normal upper limit, which all occurred in patients with heterozygous genotype (2037 TC and 2237 GA genotype). Creatine kinase (CK) did not increase significantly in any patient.

## Discussion

To our knowledge, this is the first study to explore the associations between *EL* polymorphisms and the lipid-lowering effects of rosuvastatin in CAD patients. Our present study revealed that *EL* 2037 T/C polymorphism was associated with lipid levels, but *EL* 2037T/C and 2237 G/A polymorphisms did not influence the lipid-lowering effects of rosuvastatin.

Because the view of cholesterol accumulative effect was put forward, lipid-lowering therapy has been accepted as the key preventive measure for atherosclerosis disease. Thus, statins have been widely used clinically

Rosuvastatin, one of the competitive and selective inhibitor of the HMG-CoA reductase, is widely used in the treatment of CAD and hypercholesterolaemia [19]. Previous studies have revealed that it can affect the lipids synthesis and reduce the adverse cardiovascular events [20]. The therapeutic dose of statins is relatively small in Asians, compared with Caucasian. In the present study, all CAD patient accepted 10mg rosuvastatin therapy for four to eight weeks. After treatment, the serum levels of TC, LDL-C and TG all decreased significantly (*P* < 0.05). Although the HDL-C levels also increased, it did not become statistically significant (1.07 ± 0.30 mmol/l vs.1.08 ± 0.26 mmol/l, *P* = 0.653).

EL plays an important role in the regulation of lipid metabolism, especially HDL-C, which was confirmed by several studies. By reducing the HDL-C levels and inducing the macrophages to take up native LDL-C, EL increases the susceptibility of CAD. In recent years, Sun et al. revealed that plasma EL activity was not only associated with plasma HDL-C levels, but also the risk of CAD [13]. *EL* gene is located on chromosome 18q21.1. In the past ten years, several studies have investigated the effect of the *EL* gene polymorphisms on lipid levels and found that *EL* polymorphisms were associated with lipids levels and the risk of CAD in different populations. *EL* 2037T/C and 2237G/A polymorphisms, which have been investigated by several studies, are located in the coding region of the *EL* gene. In 2003, Yamakawa-Kobayashi K et al*.* [15] revealed that *EL* 2237G/A polymorphism was associated with HDL-C levels in Japanese school-aged children. Carriers with AA genotype had lower HDL-C levels than those with GG or AA genotypes. But no studies have been conducted to investigate the relationship between the *EL* polymorphisms and lipid-lowering effects of statins until now. Given the importance of the *EL* polymorphisms in lipid metabolism, we examined the association between the *EL* 2037 T/C and 2237 G/A polymorphisms and the lipid response to rosuvastatin in Chinese patients with CAD. We identified the genotypic frequencies of *EL* 2037 T/C and 2237G/A polymorphisms in Chinese patients with CAD in this study. For *EL* 2037T/C polymorphism, the frequencies of TT, TC, and CC genotypes were 56.20, 38.84 and 4.96 % respectively. For *EL* 2237 G/A polymorphism, the frequencies of the GG, GA and AA genotypes were 38.02, 49.59, and 12.40 %, respectively. The distributions of *EL* 2037 T/C and 2237 G/A genotypes were not significantly different between Chinese CAD patients and Japanese school-aged children. In this study, we investigated the relationship between *EL* polymorphisms and lipid levels. Because the number of patients with CC genotype was small, we combined the CC and CT genotype as the C allele carrier (CC + CT). We found that the carriers with 2037 C allele had lower LDL-C levels than those with TT genotype (2.60 ± 0.74 mmol/l vs*.* 2.90 ± 0.87 mmol/l, *P* = 0.047). As for *EL* 2237 G/A polymorphism, no significant association was found between genotypes and lipid levels.

In this study, 61.98 % patients attained the guideline LDL-C levels after treatment with 10mg rosuvastatin. Whereas, no significant difference was found between *EL* polymorphisms and the percentage of patients attaining the guideline LDL-C levels.

Pharmacogenomic studies show that there is a considerable inter-individual variation in drug effects due to genetic difference. Recently, the studies on the genes, which might affect the lipid-lowering effects of statins, have mainly focused on metabolism enzymes, transporters and receptors of drugs. In our study, we did not find any significant association between *EL* genotypes and the change of lipid levels after treatment with rosuvastatin,. The negative results might be due to the possibility that the polymorphisms or expression of EL does not affect the metabolic enzyme and the substrate of statins.

Pharmacogenomic studies also showed that SNPs might affect the drug side effects, except the lipid-lowering effects of statins. The main side effects of statins are liver damage and myopathy. In our study, the ALT increased significantly in three cases. All of them happened in patients with heterozygous genotypes. The CK did not increase significantly in any patients. These suggested that the dosage of 10mg rosuvastatin in Chinese population was safe.

Some limitations of this study should be considered when we interpreted the results. Firstly, the major limitation of this study is the small number of patients. Only 121 cases were enrolled in this study and thus the numbers of genotypes were relatively small, especially *EL* 2037 CC genotype (*n* = 6). Therefore, large sample and multi-center studies are needed to confirm the results. Secondly, all of the patients of this study were selected from CAD population. The association between *EL* polymorphisms and lipid-lowering effects of rosuvastatin was not investigated in non-CAD patients with hypercholesterolemia. Our study might have the selection bias. Thirdly, the present study was a retrospective cohort study and the efficiency was limited. Finally, the follow-up time in this study was only four to eight weeks, and it is important to know whether these polymorphisms influence the long-term lipid-lowering effects of rosuvastatin.

## Conclusion

The present results indicated that the *EL* gene polymorphism might not be a pharmacogenetic marker for lipid-lowering therapy.

## Additional files

Additional file 1: Table S1. Comparison of lipid levels before and after treatment among the *EL* genotypes. (DOC 48 kb) Additional file 2: Raw data. (XLS 47 kb)
