# Supplementary material for: Endothelial lipase genetic polymorphisms and the lipid-lowering response in patients with coronary artery disease on rosuvastatin
Source: Lipids Health Dis. 2016 Sep 6;15(1):148. doi: 10.1186/s12944-016-0295-3 (PMC5012091; doi:10.1186/s12944-016-0295-3)
Supplement: Additional file 1: Table S1. — Comparison of lipid levels before and after treatment among the EL genotypes. (DOC 48 kb) [file 12944_2016_295_MOESM1_ESM.doc]

**Supplement Table 1. Comparison of lipid levels before and after treatment among the *EL* genotypes**

| **SNPs** | **N** |  | **TC^*^**  **(mmol/l)** | **TG**  **(mmol/l)** | **HDL-C**  **(mmol/l)** | **LDL-C**  **(mmol/l)** |
| --- | --- | --- | --- | --- | --- | --- |
| **2037 T/C** |  |  |  |  |  |  |
| TT | 68 | before | 4.74± 1.00 | 1.98± 1.17 | 1.08± 0.25 | 2.90± 0.87 |
|  |  | after | 3.52± 0.83 | 1.69± 1.27 | 1.08± 0.27 | 1.83± 0.70 |
|  |  | *P* | <0.001 | 0.011 | 0.931 | <0.001 |
| TC | 47 | before | 4.47± 0.88 | 2.08± 1.27 | 1.09± 0.36 | 2.64± 0.74 |
|  |  | after | 3.48± 0.85 | 1.66± 1.03 | 1.10± 0.23 | 1.79± 0.52 |
|  |  | *P* | <0.001 | 0.020 | 0.758 | <0.001 |
| CC | 6 | before | 3.92± 0.85 | 1.91± 0.57 | 0.82± 0.18 | 2.32± 0.78 |
|  |  | after | 2.92± 0.57 | 1.78± 0.84 | 0.85± 0.20 | 1.46± 0.41 |
|  |  | *P* | 0.010 | 0.715 | 0.438 | 0.010 |
| **2237 G/A** |  |  |  |  |  |  |
| GG | 46 | before | 4.63± 1.08 | 1.81± 0.95 | 1.04± 0.28 | 2.87± 0.93 |
|  |  | after | 3.55± 0.96 | 1.70± 1.18 | 1.05± 0.20 | 1.95± 0.75 |
|  |  | *P* | <0.001 | 0.478 | 0.815 | <0.001 |
| GA | 60 | before | 4.60± 0.85 | 2.21± 1.33 | 1.11± 0.33 | 2.69± 0.75 |
|  |  | after | 3.45± 0.70 | 1.70± 1.26 | 1.11± 0.28 | 1.69± 0.50 |
|  |  | *P* | <0.001 | <0.001 | 0.924 | <0.001 |
| AA | 15 | before | 4.45± 1.05 | 1.85± 1.16 | 1.03± 0.24 | 2.81± 0.80 |
|  |  | after | 3.35± 0.92 | 1.55± 0.59 | 1.04± 0.32 | 1.71± 0.58 |
|  |  | *P* | <0.001 | 0.339 | 0.927 | <0.001 |

SNPs, single nucleotide polymorphisms; TC^*^, total cholesterol; TG, triglyceride; HDL-C, high density lipoprotein cholesterol; LDL-C, low density lipoprotein cholesterol; N, number; Data are shown as mean±SD.
